# Neuropathogenic *Escherichia coli* K1 does not exhibit proteolytic activities to exert its pathogenicity

**DOI:** 10.1186/1477-5751-12-8

**Published:** 2013-05-01

**Authors:** Junaid Iqbal, Mehak Rajani, Ruqaiyyah Siddiqui, Naveed Ahmed Khan

**Affiliations:** 1 Department of Biological and Biomedical Sciences, Aga Khan University, Karachi, Pakistan; 2 Dow Medical College, Dow University of Health Sciences, Karachi, Pakistan

**Keywords:** *E. coli* K1, Protease, Collagen, Gelatin, Zymography, BSA and IgG

## Abstract

**Background:**

Proteases are well-known virulence factors that promote survival, pathogenesis and immune evasion of many pathogens. Several lines of evidence suggest that the blood–brain barrier permeability is a prerequisite in microbial invasion of the central nervous system. Because proteases are frequently associated with vascular permeability by targeting junctional proteins, here it is hypothesized that neuropathogenic *Escherichia coli* K1 exhibit proteolytic activities to exert its pathogenicity.

**Methods:**

Zymographic assays were performed using collagen and gelatin as substrates. The lysates of whole *E. coli* K1 strain E44, or *E. coli* K-12 strain HB101 were tested for proteolytic activities. The conditioned media were prepared by incubating bacteria in RPMI-1640 in the presence or absence of serum. The cell-free supernatants were collected and tested for proteases in zymography as mentioned above. Additionally, proteolytic degradation of host immune factors was determined by co-incubating conditioned media with albumin/immunoglobulins using protease assays.

**Results:**

When collagen or gelatin were used as substrates in zymographic assays, neither whole bacteria nor conditioned media exhibited proteolytic activities. The conditioned media of neuropathogenic *E. coli* K1 strain E44, or *E. coli* K-12 strain HB101 did not affect degradation of albumin and immunoglobulins using protease assays.

**Conclusions:**

Neither zymographic assays nor protease assays detected proteolytic activities in either the whole bacteria or conditioned media of *E. coli* K1 strain E44 and *E. coli* K-12 strain HB101. These findings suggest that host cell monolayer disruptions and immune evasion strategies are likely independent of proteolytic activities of neuropathogenic *E. coli* K1.

## Background

Proteases hydrolyze peptide bonds of amino acids residues in a polypeptide chain [1]. Given the presence of active residues in their catalytic sites, proteases are classified into six different types including, aspartic-, cysteine-, glumatic-, serine-, threonine- and metallo-proteases, among which serine- and metallo-proteases are most abundant in nature [1]. Besides their physiological role, many proteases are involved in pathogenesis of infectious and non infectious diseases. Among bacterial infections, two remarkable examples of proteases include lethal factor of anthrax toxin and botulinum neurotoxin produced by *Bacillus anthracis* and *Clostridium botulinum*, respectively [2,3]. The lethal factor of anthrax toxin is responsible for degradation and inactivation of host cell mitogen-activated protein kinase kinase (MAP2K) [3], whereas *Clostridium botulinium* produces a powerful neurotoxin protease that impedes acetylcholine release at peripheral nerve ending by cleaving the SNAP-25 protein. SNAP-25 is involved in vesicle fusion and facilitate acetylcholine release from axon endings into the synaptic cleft [2]. Besides the above mentioned bacterial proteases, conserved Lon, Clp, and HtrA bacterial proteases are also believed to be involved in the virulence of different Gram positive and negative bacteria [4]. Lon and Clp proteases are involved in the regulation of type III secretion system that is responsible for delivering different toxin and virulence factors to host cells. Whereas HtrA, in addition to its protease activity, also has chaperone activity which is involved in the localization and export of different virulence factors from different bacterial pathogens [4].

*E. coli* K1 is a leading cause of infant meningitis and sepsis in both developed and developing world. These infections have high mortality rates of 40-50% and affect 5–50 infants among 100,000 live births and estimated to be responsible for ~50,000 deaths worldwide per year [5-8]. One reason for such high mortality rate is inadequate understanding of pathogenesis and the pathogen itself. A number of virulence factors including cytotoxic necrotizing factor 1 (CNF1), FimH, outer membrane protein A (OmpA), Ibe proteins, TraJ, and As1A have been identified [9], but the role of proteases in *E. coli* K1 pathogenesis have not been studied. Given that proteases are frequently associated with vascular permeability [1,10], here it is hypothesized that the neuropathogenic *E. coli* K1 exhibit proteolytic activities to exert its pathogenicity.

## Materials and methods

*E. coli* K1 strain E44, a spontaneous rifampin-resistant mutant of a cerebrospinal fluid isolate of K1-encapsulated *E. coli* RS218 (O18:K1:H7) [11] was used as an invasive isolate, while *E. coli* K-12 strain HB101 was used a non-invasive laboratory isolate in the present study. For routine culturing, both bacteria were grown in Luria–Bertani (LB) broth overnight. For zymographic assays, bacteria were grown overnight with shaking under aerobic condition at 37°C in RPMI 1640. Next day the optical density was adjusted to 0.22 for E44 and 0.35 for HB101 using 595 nm wavelength yielding approximately 1 × 10^8^ per mL bacterial colony forming units (c.f.u.). To determine proteolytic activities, whole cell lysates were prepared by incubating various bacterial counts in 2× SDS sample buffer without beta-mercaptoethanol and kept unboiled for 30 min at room temperature, followed by vortexing. Finally, bacterial lysates were tested for proteases in zymography. For positive controls, *Acanthamoeba castellanii* lysates were prepared. Briefly, amoebae (10^4^ parasites in 10 μL) were incubated in lysis buffer as above and tested for proteolytic activities in substrate zymography.

To determine the presence of extracellular proteolytic activities, *E. coli* conditioned media were prepared. To produce conditioned media, *E. coli* K1 and K12 were grown overnight with shaking under aerobic condition at 37°C in RPMI 1640 with or without 10% serum fetal calf serum. The cell-free conditioned media was removed by centrifugation at 10,000 × *g* for 2 min and 10 μl of these were loaded along with uninoculated medium on SDS-PAGE gels containing gelatin and collagen as substrates described below.

For zymographic assays, whole cell bacterial lysates or their conditioned media were mixed (1:1) with sample buffer (containing 4% sodium dodecyl sulfate (SDS) but without β-mercaptoethanol) and electrophoresed on 7.5% SDS-polyacrylamide gel electrophoresis (SDS-PAGE) containing gelatin (obtained from bovine skin, Sigma-Aldrich; 1 mg/mL final conc.) or collagen I (obtained from rat tail, Sigma-Aldrich; 1 mg/mL final conc.). After electrophoresis, gels were soaked in 2.5% Triton X-100 (w/v) solution for 120 min to remove SDS. Finally, the gels were incubated in a developing buffer (50 mM Tris–HCl, pH 7.5, containing 5 mM CaCl2, 100 mM NaCl, 0.5 mM MgCl2, and 0.002 mM ZnCl2) at 37°C overnight, rinsed, and stained with Coomassie brilliant blue. Areas of gelatin digestion were visualized as non-staining regions in the gel. To determine the effects of various pH on *E. coli* proteolytic activity, cell-free *E. coli* K1 conditioned medium was electrophoresed, and gels were incubated overnight in pH 3, 4, 5, 6, 7, 8, 9, and 10 (0.1 M citrate buffer was used to adjust pH 3, 4, and 5; 0.1 M phosphate buffer was used for pH 6, 7 and 8; and 0.2 M glycine buffer was used for pH 9 and 10). Following this incubation, gels were stained with Coomassie brilliant Blue. The results are representative of three independent experiments.

For protease assays, *E. coli* conditioned media (10 μL) were incubated with 2 μg of bovine serum albumin (BSA) and rabbit IgG (rIgG) in a final volume of 20 μL in incubation buffer (50 mM Tris-Cl pH 8.0, 100 mM NaCl, 5 mM CaCl_2_, 500 μM MgCl_2_ and 2 μM ZnCl_2_) in 0.5 mL eppendorf tubes. Tubes were incubated for 18 h at 37°C. The BSA and rIgG in the absence of conditioned media were used as negative controls, while *A. castellanii* conditioned media (known to produce proteases) [12], were used as positive controls. Following this incubation, samples were mixed with equal volume of 2× SDS loading buffer, boiled and loaded onto 12% SDS-PAGE gel to determine BSA/rIgG degradation. After electrophoresis gels were stained with Coomassie Brilliant Blue and visualized. The results are representative of three independent experiments.

## Results and discussion

**Figure 1:**
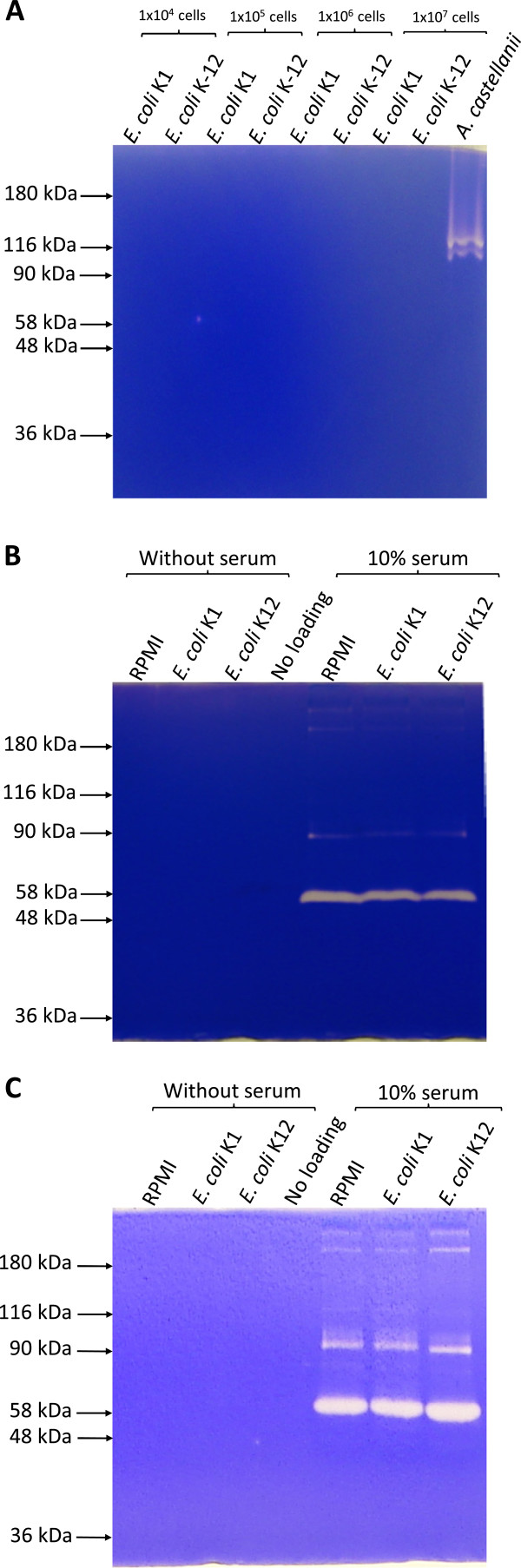
***E. coli *K1 did not exhibit proteolytic activities in zymography. (A)** Neuropathogenic *E. coli* K1 strain E44 and *E. coli* K-12 laboratory strain HB101 were grown overnight with shaking under aerobic condition at 37°C in RPMI 1640. Next day the optical density was adjusted to 0.22 for K1 and 0.35 for K-12 using 595 nm wavelength yielding approximately 1 × 10^8^ per mL bacterial c.f.u. Various bacterial counts were mixed with lysis buffer and loaded onto 7.5% SDS-PAGE gel containing gelatin as substrate, while *A. castellanii* (10^4^ cells) was used as a positive control. After electrophoresis, gels were washed for four times with 2.5% Triton X-100 solution for 30 min and incubated overnight in developing buffer (50 mM Tris-Cl pH 7.5, 100 mM NaCl, 5 mM CaCl_2_, 500 μM MgCl_2_ and 2 μM ZnCl_2_). Following this incubation, gels were stained with Coomassie Brilliant Blue, destained and finally visualized. **(B and C)***E. coli* K1 and K12 were grown overnight with shaking under aerobic condition at 37°C in RPMI 1640 with or without 10% serum fetal calf serum. Next day, cells were removed by centrifugation and cell free conditioned mediums were collected and 10 μl of these were loaded along with uninoculated medium on 7.5% gelatin (**B**) and collagen (**C**) substrates zymography gels as described in A. Note that neither whole bacteria nor their conditioned medium degraded gelatin/collagen in zymographic assays tested. The results are representative of three independent experiments.

**Figure 2:**
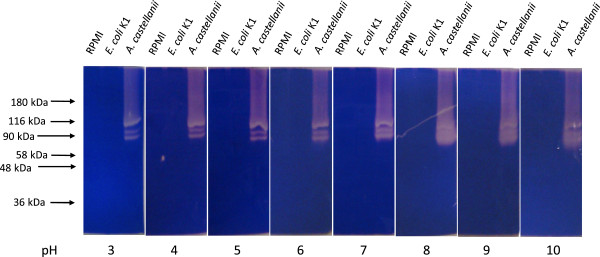
**No proteases activity detected in *E. coli *K1 conditioned medium at different pH.** Cell-free *E. coli* K1 conditioned medium was prepared as described in Figure 1A. After electrophoresis, gels were washed for four times with 2.5% Triton X-100 solution for 30 min and incubated overnight in 0.1 M citrate buffer (pH 3, 4 and 5) or 0.1 M phosphate buffer (pH 6, 7 and 8) or 0.2 M glycine buffer (pH 9 and 10). All buffers were added with 100 mM NaCl, 5 mM CaCl_2_, 500 μM MgCl_2_ and 2 μM ZnCl_2_. Following this incubation, gels were stained with Coomassie Brilliant Blue and representative zymographs were visualized. Note that *E. coli* K1 conditioned medium does not exhibit protease activity at any pH ranging from 3–10. The conditioned medium of *Acanthamoeba castellanii* was used as a positive control.

To determine the proteolytic activities of *E. coli* whole cells lysates or their conditioned media, zymographic assays were performed. The lysates of *A. castellanii* (positive control) exhibited gelatin degradation (Figure 1A); however, neither *E. coli* K1 strain E44 lysates nor *E. coli* K-12 strain HB101 tested, showed gelatin degradation in zymographic assays (Figure 1A). Similarly, the conditioned medium of *E. coli* did not exhibit gelatin or collagen degradation in zymographic assays (Figure 1B and 1C). As expected, serum alone exhibited proteolytic activities (Figure 1B and 1C). The conditioned medium prepared in the presence of 10% foetal calf serum did not show any additional noticeable protease band on zymograms (Figure 1B and 1C). When higher percentages zymography gels (10% and 12%) were used to detect low molecular mass protease(s), no additional protease bands on zymograms were observed (data not shown). When tested under various pH, the results revealed that *E. coli* K1 conditioned medium does not exhibit protease activity at any pH ranging from 3–10 (Figure 2).

**Figure 3:**
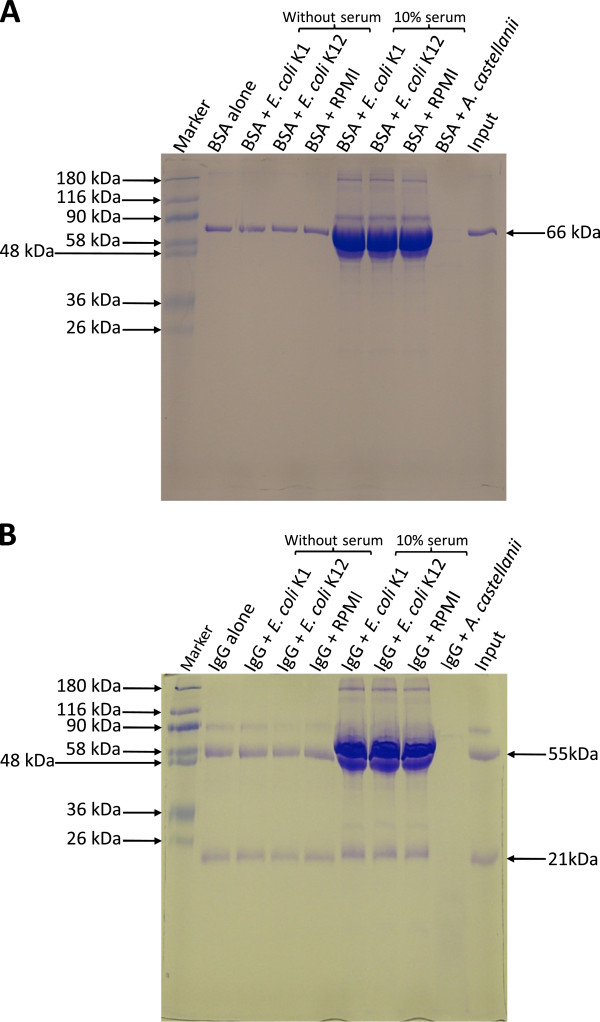
***E. coli *K1 did not affect degradation of bovine serum albumin (BSA) and rabbit immunoglobulin G (rIgG) in protease assays.** The conditioned medium of *E. coli* K1 strain E44, *E. coli* K-12 strain HB101 and *A. castellanii* was prepared as described in Materials and Methods. The conditioned media were incubated with 2 μg of BSA **(A)** and rIgG **(B)** overnight in incubation buffer (50 mM Tris-Cl pH 8.0, 100 mM NaCl, 5 mM CaCl_2_, 500 μM MgCl_2_ and 2 μM ZnCl_2_) at 37°C. Next day, an equal volume of 2× SDS sample loading buffer was added in all samples, boiled and loaded onto 12% SDS-PAGE gel. After electrophoresis gels were stained with Coomassie Brilliant Blue and visualized. Note that bacterial conditioned media did not degrade BSA/rIgG in protease assays tested. The results are representative of three independent experiments.

Furthermore, *E. coli* K1 proteases did not exhibit substrate specificity as no proteolytic activity was observed on gelatin or collagen substrate SDS-PAGE gels. To determine whether assay conditions may have affected extracellular proteases of *E. coli*, protease assays were performed by incubating conditioned medium with BSA and rIgG, followed by electrophoresis on SDS-PAGE gels. The findings revealed that the conditioned medium of neither *E. coli* K1 nor *E. coli* K-12 had any effect on the degradation of BSA and rIgG. Conversely, the conditioned medium of *A. castellanii* (known to possess a number of proteases) [12] degraded both of these substrate proteins completely (Figure 3). Similar results were observed when human secretary IgA was used as a substrate in the protease assays (data not shown).

The proteolytic enzymes have been identified as virulence factors in various bacterial pathogens including *Vibrio vulnificus*[13], *Pseudomonas aeruginosa*[14], *Staphylococcus aureus*[15], *Porphyromonas gingivalis*[16], and their role as therapeutic target is under intense investigations. Given the role of proteases as protein quality control machinery as well as controlled proteolysis of regulatory proteins, highlights their involvement in bacterial viability and virulence. For example, Clp protease complexes play a vital role in regulating virulence factor(s) production in Gram-positive bacteria, while conserved proteases of Gram negative bacteria impacting biological processes across the bacterial envelope affecting bacterial viability and pathogenesis [4]. Genomic analysis of *E. coli* K-12 revealed that it encodes for more than 70 proteases [17] and studies have reported the presence of cellular and secreted proteases from different *E. coli* strains [18,19] but under the assays employed and the experimental conditions tested, no proteases were detected in either neuropathogenic *E. coli* K1 strain E44 or *E. coli* K-12 laboratory strain HB101, tested in the present study. In addition to strain differences, a likely explanation of these contradictory findings is that the proteolytic activity is inducible (expressed in response to milk proteins or other environmental conditions) and limited constitutive proteases. Future studies are needed to grow *E. coli* K1 under a variety of conditions including different oxygen tensions, heat shock conditions as well as in the presence of different host cells to establish extracellular proteolytic activities of neuropathogenic *E. coli* K1.

## Competing interests

The authors declare that they have no competing interests.

## Authors’ contributions

NK conceived the study. JI and RS designed and conducted all experiments under the supervision of NAK. RS, JI, MK and NAK contributed to the writing of the manuscript. All authors approved the final manuscript.
